# The impact of tournament incentives on corporate credit repair: Evidence from China

**DOI:** 10.1371/journal.pone.0340063

**Published:** 2026-01-29

**Authors:** Jianxiu Wang, Yuntian You, Yuanxiang Dong, Rongwang Guo

**Affiliations:** 1 School of Management Science and Engineering, Shanxi University of Finance and Economics, Taiyuan, China; 2 School of Economics and Management, Taiyuan University of Technology, Taiyuan, China,; 3 School of Agricultural Economics and Management, Shanxi Agricultural University, Jinzhong, China; Yamanashi Gakuin University: Yamanashi Gakuin Daigaku, JAPAN

## Abstract

This study investigates the impact of tournament incentives on corporate credit repair and re-repair. Drawing on tournament and agency theory, respectively, we argue that tournament incentives improve corporate credit repair and re-repair by motivating non-CEO executives'  effort and risk-taking, and by inducing incumbent CEOs to supervise subordinates to restrain opportunistic behavior. Using data from Chinese listed companies from 2009 to 2023 and employing SHAP values and benchmark traditional econometric methods, our results show that tournament incentives have a positive impact on corporate credit repair and re-repair. Furthermore, CEO shareholding strengthens the positive impact of tournament incentives on corporate credit repair, whereas firm age weakens the positive impact of tournament incentives on corporate credit repair. Additionally, firm size and leverage weaken the positive impact of tournament incentives on corporate credit re-repair. This paper sheds light on the role of tournament incentives on corporate executives for policymakers to enhance corporate credit repair.

## 1 Introduction

Corporate dishonesty refers to the violation of the principle of integrity, non-compliance with contractual obligations, fraudulent activities, or other deceptive practices occurring during business operations [1]. Such behaviors erode stakeholders' trust [2], undermine investor confidence [3], precipitate operational inefficiencies [4], and heighten risks of bankruptcy and delisting [5]. Existing studies have thoroughly investigated the antecedents of corporate dishonest behavior through compensation incentives [6–8], executive team characteristics [9,10], board characteristics [11–13], executive backgrounds [13], equity structure [14], and other institutional factors. However, significant gaps remain in understanding the post-breach remediation mechanisms—specifically, how defaulting enterprises can achieve credit repair and, in many cases, repeated repair. Corporate credit repair refers to the formalized process through which a firm, within a designated period and under prescribed conditions and procedures, actively remedies prior breaches of trust, thereby becoming eligible to expunge the negative effects of its misconduct from regulatory credit assessments [15]. This issue is particularly critical in the context of an emerging and transitional economy like China. The Chinese government has been actively constructing a nationwide social credit system, where a firm's credit record directly impacts its market access, financing capacity, and regulatory scrutiny. Furthermore, within China's relation-based business society, reputation serves as a critical informal governance mechanism, a damaged credit record severely hampers a firm's ability to build and maintain essential business and government relationships. According to data published by China's State Administration for Market Regulation, by the end of 2020, there were 6.6486 million enterprises on the abnormal operation list and 0.9824 million on the serious illegal and dishonest enterprise list [16]. Thus, understanding the mechanisms driving both initial credit repair and subsequent re-repair is of urgent concern to both corporate stakeholders and Chinese policymakers.

Executive compensation incentives play a pivotal role in mitigating agency problems by aligning managerial objectives with shareholder interests [7,8]. Designing optimal executive compensation schemes aligned with company performance can incentivize managers to pursue maximizing shareholder value rather than their own goals [17–19]. While the majority of executive compensation research investigates performance-based incentives, another stream examines rank-order or tournament-based incentives (also known as tournament incentives) [19]. Tournament theory views executives as competitors vying for promotion. In this context, the pronounced compensation differential between the CEO and subordinate executives constitutes the prize of tournament [18–20], incentivizes executives to outperform peers, exert greater effort, and enhance both promotion prospects and organizational performance. Previous studies indicate tournament incentives could improve corporate performance [18], reduce debt cost and default risk [21], diminish instances of corporate financial misconduct [8], reduce the occurrence of both core and non-core financial restatements [22], and improve innovation efficiency [23]. However, tournament incentives can also yield negative effects. For instance, prior research has found that tournament incentives are associated with greater performance misreporting [24], more sabotage activities [25], higher likelihood of fraud [7], and greater cash flow uncertainty [26]. Regarding corporate credit, extant literature examined the influence of tournament incentives on corporate dishonest behavior [7,8,22]. Given the opaque nature of corporate misconduct, its detection remains inherently challenging [27]. Consequently, firms that have ostensibly undergone credit repair may nonetheless relapse into misconduct. Thus, an important and underexplored question persists—can tournament incentives promote not only initial credit repair but also subsequent re-repair among previously dishonest firms?

To confirm the relationship between tournament incentives and corporate credit repair as well as re-repair, we draw upon tournament theory [19] and agency theory [28] to develop a theoretical model explicating the effect of tournament incentives on corporate credit repair and re-repair. Specifically, a pronounced pay differential between the CEO and the median compensation of subordinate senior executives may compel non-CEO executives to augment their effort and engage in higher-risk initiatives in pursuit of upward mobility, thereby enhancing their prospects of attaining the top position [18]. However, such incentive mechanisms may also encourage engagement in opportunistic or unethical behaviors as a strategy to outperform rivals in the internal promotion tournament [29]. This not only contravenes the current CEO' s interests but also imperils their position, security, and social status. To ensure security of position, compensation, and welfare, the current CEO, motivated by self-interest [28], will actively supervise and prevent other top executives' negative efforts in the internal promotion tournament [30]. Concurrently, significant differences manifest in the performance of untrustworthy companies before and after the process of credit repair and re-repair. To safeguard their own standing and maximize their tournament positioning, CEOs will strategically implement measures to foster corporate credit repair and re-repair. Moreover, we investigate how firm age and CEO shareholding moderate the effect of tournament incentives on corporate credit repair. Besides, given that the cost of corporate credit re-repair significantly exceeds that of initial credit repair, the circumstances encountered by corporations are inherently more intricate. Hence, we incorporate firm size and leverage as moderating variables to analyze their moderating effect on the relationship between tournament incentives and corporate credit re-repair. We argue that CEO shareholding strengthens the positive impact of tournament incentives on corporate credit repair. Conversely, firm age weakens the positive impact of tournament incentives on corporate credit repair. Additionally, firm size and leverage weaken the positive impact of tournament incentives on corporate credit re-repair. Based on the data of Chinese listed companies from 2009 to 2023, using the SHAP values and benchmark traditional econometric methods, we empirically validate our arguments.

This study contributes to the extant literature in three primary ways. First, whereas previous research on tournament incentives has primarily centered on the impact on corporate performance [18], risk-taking [31], and fraud [7], our research pivots to explore the impact of tournament incentives on corporate credit repair and re-repair. This study not only extends the theoretical boundaries of tournament theory but also deepens our comprehension of how corporate compensation policies intricately intertwine with corporate credit repair and re-repair. Second, while existing scholarship has predominantly investigated the antecedents of corporate dishonesty [7,32–34], overlooking the resolution of such behaviors post-occurrence. Our research sheds light on this overlooked aspect by providing empirical evidence of the positive influence of tournament incentives on corporate credit repair and re-repair, thereby enriching the research in the domain of corporate credit literature. Third, our research unveils the moderating roles of firm age and CEO shareholding in the relationship between tournament incentives and corporate credit repair, as well as the moderating role of firm size and leverage in the relationship between tournament incentives and corporate credit re-repair, thereby further clarifying the boundary conditions of the relationship between tournament incentives and corporate credit repair and re-repair in the context of China's evolving institutional and regulatory environment.

## 2 Literature review

The substantial pay gap between the CEO and non-CEO executives has long challenged traditional economic explanations [35]. To address this puzzle, Lazear and Rosen (1981) introduced the tournament theory, which conceptualizes non-CEO executives as competitors in an internal contest for promotion. Within this framework, the substantial pay differential between the CEO and non-CEO executives functions as a prize for the tournament winner, thereby providing strong motivational incentives for highly qualified managers to exert greater effort [36]. Previous research on tournament incentives indicates that empirically disentangling the underlying drivers of the relation between tournament incentives and corporate behavior and performance. Drawing on the intra-firm tournament incentive, the substantial gap between the CEO's compensation and the median pay of the next level of senior officers in the firm constitutes a more substantial incentive. This motivates second-tier executives to exert greater effort, undertake higher-risk initiatives to maximize outcomes, and increase their chances of securing the top position [18]. Additionally, scholars have also ascertained that tournament incentives could reduce the cost of debt and default risk [21], corporate financial misconduct [8], the occurrence of both core and non-core financial restatements [22], and are more likely to enhance innovation efficiency [23].

However, despite abundant empirical evidence suggesting the positive impact of tournament incentives, their overall empirical implications remain contested. Some studies even revealed the adverse impacts of tournament incentives on corporate behavior and performance. The large pay gap between CEOs and non-CEO executives can entrench CEOs, enabling them to exert greater influence over pay-setting processes and expropriate shareholder wealth [37]. Goel and Thakor (2008) demonstrated that tournament incentives lead senior managers to take greater risks to enhance their prospects for advancement to the CEO position. Supporting this notion, Kini and Williams (2012) further documented a positive association between firm risk and promotion-based incentives, and that the mechanisms through which promotion-based incentives affect corporate risk include acquisitions, R&D intensity, company focus, and leverage. Similarly, tournament-driven promotion incentives have been linked to heightened risk of corporate fraud [7], negative effort leading to legal disputes [29], and risk-enhancing financing decisions that increase cash flow volatility [26].

While increasing scholarly attention has been directed toward the magnitude and composition of CEO compensation relative to other top executives and industry benchmarks, relatively few studies have investigated the link between tournament incentives and corporate credit. Recent research has examined the impact of tournament incentives on corporate dishonest behavior. For instance, Haß et al. (2015) demonstrated that firms with strong tournament incentives exhibited a greater propensity to engage in fraud activities. Similarly, Zhong et al. (2021) found that tournament incentives could inhibit corporate financial misconduct. These findings highlight the argument that tournament incentives have an impact on corporate dishonest behavior. Nevertheless, these studies primarily concentrate on whether such incentives act as deterrents or catalysts for dishonest behavior, neglecting to explore their potential role in facilitating credit repair among previously untrustworthy firms. Importantly, the sizable financial gains often derived from corporate dishonesty may incentivize firms in the process of credit re-repair to re-engage in misconduct, thus complicating the recovery process. Therefore, we posit that the impact of tournament incentives on corporate credit repair and re-repair represents a pertinent empirical question that merits investigation and contributes to this emerging literature.

## 3 Theoretical model and research hypotheses

Corporate dishonest behavior undermines the company's reputation, increases financing costs, lowers employee morale, results in market share, legal proceedings and penalties, etc., ultimately diminishing the company's output level. Based on this, this study assumes that enterprise output obeys the Cobb-Douglas (C-D) production function and incorporates managerial efficiency as a key input in the model. This premise rests on the principle that higher levels of management efficiency enhance firm output [38]. In addition, untrustworthy companies will engage in credit repair efforts, during which they are likely to cultivate more robust relationships with stakeholders, including customers and societal actors. Such efforts can, in turn, boost the firm's output level, thereby incorporating corporate creditworthiness ${R_{t}}^{i}$ ($0<{R_{t}}^{i}<1$) into the model. Consequently, the enterprise output function can be expressed as:


$$Y_{t}^{i}={AR}_{t}f(L,K,M)=A_{t}R_{t}^{i}{\left(L_{t}^{i}\right)}^{\alpha }{\left(K_{t}^{i}\right)}^{\beta }{\left(M_{t}^{i}\right)}^{\theta },\;0<\alpha ,\beta ,\theta <1,\;\alpha +\beta +\theta =1$$ (1)

Among them, ${Y_{t}}^{i}$ represents the output level of the ith company in period *t*; $R_{t}$ represents enterprise's credit level, L,K,M represen*t* the labor, capital, and management efficiency factors of the ith enterprise in period *t*; $A_{t},\alpha ,\beta ,\theta$ respectively represents the corresponding production technology, labor factor output elasticity, capital fac*t*or output elasticity, and management efficiency factor output elasticity of the enterprise in period *t*.

Then, the target profit function of the ith enterprise in period t can be expressed as follows:


$$\pi =P_{t}{Y_{t}}^{i}-w_{t}{L_{t}}^{i}-r_{t}{K_{t}}^{i}-\lambda_{t}{M_{t}}^{i}=P_{t}A_{t}R_{t}({L_{t}}^{i}{)}^{\alpha }({K_{t}}^{i}{)}^{\beta }({M_{t}}^{i}{)}^{\theta }-w_{t}{L_{t}}^{i}-r_{t}{K_{t}}^{i}-\lambda_{t}M_{t}$$ (2)

Among them, $P_{t}$ represents the sales price of the enterprise's output in a perfectly competitive market, $w_{t}$ is the wage of the hired labor factor in the labor market, $r_{t}$ is the capital price in the capital factor market, $\lambda_{t}$ is the price of the management efficiency factor in the market, the price is determined by factors such as the level of compensation shareholders are willing to give managers.

By computing the first-order partial derivative with respect to the labor input, and recognizing that in a perfectly competitive market factor prices equate to marginal products $\lambda_{t}=MP{M_{t}}^{i}$, we obtain:


$$P_{t}A_{t}\theta R_{t}({L_{t}}^{i}{)}^{\alpha }({K_{t}}^{i}{)}^{\beta }({M_{t}}^{i}{)}^{\theta -1}=\lambda_{t}$$ (3)

By transforming Eq (3), we can derive the theoretical relationship between corporate creditworthiness in terms of managerial efficiency, labor, and capital.


$$R_{t}=\frac{\lambda_{t}({M_{t}}^{i}{)}^{1-\theta }}{P_{t}A_{t}\theta ({L_{t}}^{i}{)}^{\alpha }({K_{t}}^{i}{)}^{\beta }}$$ (4)

Eq (4) illustrates that there is a positive correlation between the enterprise creditworthiness and the price of management efficiency factors. In addition, from α+β+θ=1 in formula (1), we can get 1−θ>0, that is, there is a positive correlation between the enterprise creditworthiness and the management efficiency factors.

On the other hand, according to the tournament theory, the substantial pay gap between CEO and non-CEO executives fosters competitive effort because the salary of the CEO incentivizes those who might become the next CEO [19]. To investigate the potential connections between corporate credit repair, re-repair, and tournament incentives, we build upon the foundational tournament model introduced by Lazear and Rosen (1981). By incorporating key aspects of corporate governance, our model seeks to explore the intricate dynamics within this theoretical framework. Consider that the enterprise is a risk-neutral enterprise, with its output depending on the efforts of its CEO competitors, that is:


$$Y=\sum_{i=1}^{n}y_{i}\left(e_{i}\right)$$ (5)

Among them, Y is the enterprise output, $y_{i}(\cdot )$ is the output function of CEO competitor *i*, and $e_{i}$ are the effort level of CEO contestants. Based on existing research [19], assuming that there are two homogeneous and risk-neutral CEO contestants *m* and *n* in the enterprise, then its output can be expressed as:


$$y_{i}=e_{i}+\varepsilon_{i}$$ (6)

Where i=m,n. $\varepsilon_{i}$ is the uncertainty factor. It can be seen from the output function of CEO contestants that their output depends on their own efforts, but is also affected by random factors. The CEO competitor who wins the output competition will be promoted to CEO and receive a superior salary *W*_*h*_, while the loser can only receive a lower salary *W*_*l*_. We denote *∆*_*W*_ = *W*_*h*_-*W*_*l*_ as the CEO pay gap, capturing the magnitude of tournament incentives. In addition, based on existing research, it is assumed that the effort level of CEO contestants will incur a cost of c(e), the cost function is $c\left(e\right)=\frac{h}{\text{2}}\cdot {e_{i}}^{\text{2}},h>0$, and satisfies: c'(e)>0,c''(e)>0.

Where *C* represents the probability that CEO contestants win the tournament. Based on the above basic assumptions, especially the basic principle that executive compensation under the competition mechanism only depends on their relative performance, the probability function of CEO contestants winning in the championship competition can be expressed as:


$$\begin{array}{cc} C & =prob(y_{m}>y_{n})\mathrm{\backslash hfill}\\ \;\;\;\;\;\;\; & =prob(e_{m}-e_{n}>\epsilon_{n}-\epsilon_{m})\mathrm{\backslash hfill}\\ \;\;\;\;\;\;\; & =prob(e_{m}-e_{n}>\xi )=G(e_{m}-e_{n})\mathrm{\backslash hfill} \end{array}$$ (7)

In Eq (7), $\xi =\epsilon_{n}-\epsilon_{m}$, ξ~g(ξ), G(·) is the distribution function of ξ, and *E*(ξ)=0, *Var* (ξ)= $2\delta^{\text{2}}$ (Because $\varepsilon_{m}$, $\varepsilon_{n}$ are independent random variables).

The expected reward of CEO contestants *i* is $C_{i}W_{h}+(1-C_{i})W_{l}$. Under the assumption of risk neutrality, the expected utility of CEO contestants *i* choosing to maximize the effort level is:


$$U_{i}=C_{i}W_{h}+(1-C_{i})W_{l}-c(e_{i})=C_{i}W_{h}+(1-C_{i})W_{l}-\frac{h}{\text{2}}\cdot e_{i}^{\text{2}}={△}_{W}C_{i}-W_{l}-\frac{h}{\text{2}}\cdot e_{i}^{\text{2}}$$ (8)

Therefore, CEO contestants *m* chooses the level of effort to maximize its expected utility. The first-order condition for utility maximization is:


$$\frac{\partial C_{m}}{\partial e_{m}}={△}_{W}g(e_{m}-e_{n})-he_{m}$$ (9)

To set Eq (9) equal zero, we get the optimal level of effort ($e_{m}$) from CEO contestant *m*,


$$e_{m}^{*}=\frac{{△}_{W}g(e_{m}-e_{n})}{h}$$ (10)

Similarly, following the above framework, we can derive the maximization condition of CEO contestants *n*,


$$e_{n}^{*}=\frac{{△}_{W}g(e_{n}-e_{m})}{h}$$ (11)

Since G(·) is the distribution function of ξ and *E*(ξ) = 0, *Var* (ξ) = $2\delta^{\text{2}}$*,* G(x)=1−G(−x), and g(x)=g(−x), then the model gets,


$$g(e_{m}-e_{n})=g(e_{n}-e_{m})$$ (12)

Therefore, under the so-called balance conditions, each CEO contestant shall choose the same effort level,


$$\;e^{*}=e_{m}^{*}=e_{n}^{*}=\frac{{△}_{W}g(0)}{h}$$ (13)

As indicated by Eq (13), a widening salary gap between the CEO and other executives amplifies the effort exerted by CEO contestants. Additionally, existing literature supports the notion that enhanced managerial incentives are associated with higher managerial effectiveness [39]. Consequently, the expansion of the salary gap can further motivate CEO contestants, thereby improving the management efficiency of the company, that is, ${M_{t}}^{i}$ ∝ *∆*_*W*_. Eq (4) indicates that enterprise creditworthiness is positively correlated with management efficiency factors, that is, $R_{t}$ ∝ ${M_{t}}^{i}$. This is because higher managerial efficiency enhances internal governance capacity [40], enabling managers to promptly identify and rectify the institutional deficiencies that have led to credit impairment. Meanwhile, efficient managers possess superior resource allocation capabilities [41], allowing them to achieve optimal investment in credit-repair-related activities under resource constraints—such as debt repayment, internal control improvement, and ESG enhancement—thereby improving the firm's operational soundness and information transparency. Furthermore, enhanced managerial efficiency strengthens corporate communication and external disclosure capabilities [42], enabling the firm to convey credible and persuasive rectification signals to investors, regulators, and the broader public, thereby accelerating the process of credit repair. Combining *∆*_*W*_ ∝ ${M_{t}}^{i}$ and $R_{t}$ ∝ ${M_{t}}^{i}$, there exists a demonstrable positive relationship between tournament incentives (pay gap) and enterprise creditworthiness, that is *∆*_*W*_ ∝ $R_{t}$. In this sense, tournament incentives may serve as a mechanism for credit repair by motivating managers of firms that have breached trust to take corrective actions to restore external confidence. Similarly, for firms with repeated breaches of trust, heightened internal competition can further encourage CEOs to strengthen governance and disclosure practices, thereby facilitating credit re-repair. Based on this reasoning, we propose the following hypothesis:

H1a: Tournament incentives have a positive impact on corporate credit repair

H1b: Tournament incentives have a positive impact on corporate credit re-repair

This study distinguishes between corporate credit repair and credit re-repair, based on their differences in governance stage, motivation, and constraining conditions. Corporate credit repair refers to the process of rebuilding after the first instance of credit loss, in which the key lies in the firm's motivation and willingness to restore external trust and internal governance capacity. Firm age represents organizational inertia and path dependence, while CEO shareholding reflects an internal governance mechanism that combines incentives and constraints. Therefore, the key to the credit repair stage lies in the transmission and supervision of incentives, making the inclusion of firm age and CEO shareholding as moderating variables theoretically justified.

Firm age influences its experience, resources, relationship with stakeholders, reputation, strategic position, and market share. Additionally, it affects strategic choices and firm performance over time [43]. On one hand, the capability-enhancing processes associated with aging can facilitate the execution of established routines [44] or enable firms to more effectively identify and exploit new technological opportunities [45]. Moreover, older firms tend to leverage their extensive business experience, leading to greater growth persistence compared to younger firms [46,47]. However, older firms might suffer from "liability of obsolescence" and "liability of senescence" [48]. This implies that the growth persistence of old firms is mitigated due to their struggles in adapting their strategies to changing business conditions, as well as increasing inertia and organizational rigidity. Besides, as firms mature, the knowledge and capabilities of their CEOs may become outdated and less adaptable to change [49]. As a result, as the age of the company continuously increases, the CEO's ability to repair corporate credit diminishes as the company faces the challenges of "liability of obsolescence" and "liability of senescence" as well as the issues encountered by the CEO themselves.

H2: Firm age weakens the positive impact of tournament incentives on corporate credit repair.

The level of CEO shareholding serves as a critical indicator of the CEO's positional power within the organization [50]. CEOs with significant ownership stakes can exert substantial influence on board of directors and shareholders, thereby aligning their strategic vision with the company's objectives [51]. Furthermore, CEOs who hold a larger proportion of company shares are more likely to prioritize the firm's long-term performance, reduce the temptation for excessive "empire-building" behaviors, and enhance operational efficiency [52]. This alignment occurs because a higher shareholding proportion directly ties the CEO's financial well-being to the company's stock performance, effectively positioning them as a major internal shareholder. As a result, both the CEO and the shareholders stand to gain from increases in stock value, yet their wealth is equally vulnerable to declines in stock price [53]. Consequently, to safeguard their personal wealth and the interests of long-term shareholders, CEOs are motivated to closely monitor the behavior of non-CEO executives and implement strategies for credit repair, thereby protecting their wealth and the interests of long-term shareholders.

H3: CEO shareholding strengthens the positive impact of tournament incentives on corporate credit repair.

Corporate credit re-repair occurs when a firm rebuilds after a repeated credit loss, and its core lies in the firm' s resource endurance and risk governance capability. Firm size reflects both resource redundancy and bureaucratic inertia, while leverage represents financial pressure and external constraints. Therefore, in the stage of corporate credit re-repair, using firm size and leverage as moderating variables is theoretically appropriate, as they capture how resource and risk constraints condition the effectiveness of tournament incentives.

In the process of corporate credit re-repair, it is essential for CEOs to maintain centralized authority to ensure the effective implementation of this strategy. However, as the scale of the company continues to expand, the concentration of the CEO's power tends to diminish; Moreover, firm size is closely linked to the complexity of its organizational structure [54]. Smaller firms typically exhibit flatter hierarchies, fewer senior executives, and more centralized decision-making authority [55]. Consequently, CEOs in smaller firms are more capable of exercising oversight, overseeing the behavior of non-CEO executives, and implementing measures to address corporate credit re-repair. In contrast, large firms possess more complex hierarchies and a greater number of non-CEO executives, which can dilute the CEO's power and reduce their influence over decision-making [56]. Furthermore, larger firms are more likely to experience inertia effects that may outpace the managerial discretion available, thereby weakening the CEO's ability to steer corporate decisions [57]. Therefore, compared to smaller firms, CEOs in larger organizations encounter increased difficulties in monitoring the dishonest behavior of non-CEO executives and in implementing effective credit re-repair strategies. As a result, the CEO's role as a monitoring mechanism diminishes [30], and their ability to enforce credit re-repair actions is increasingly constrained as firm size increases.

H4: Firm size weakens the positive impact of tournament incentives on corporate credit re-repair.

The influence of firm leverage on investment decisions is a critical topic in corporate finance. A high leverage ratio can undermine a firm's financing capacity [58], elevate bankruptcy costs, and lead to inefficiencies [59]. Additionally, a high leverage ratio is typically negatively correlated with corporate investment [60]. When leverage is high, companies face heightened financial risks [61], which could increase uncertainty and impede the capacity to engage in effective credit re-repair. Moreover, in a high-leverage scenario, a firm's primary financial goal becomes focused on debt reduction, improving asset quality, and optimizing capital efficiency, often necessitating more conservative financial strategies and curtailing non-essential expenditures and investments. However, the resources required for credit re-repair in the case of past corporate missteps are likely to be significantly greater than those needed for initial credit repair, complicating the re-repair process. In contrast, tournament incentives usually motivate senior managers to pursue more aggressive strategies to improve company performance for career advancement or additional rewards. Yet, these incentives often come with increased costs and risks [31]. In the context of high leverage, these additional risks could exacerbate a company's difficulty in achieving successful credit re-repair. Therefore, leverage weakens the positive impact of tournament incentives on corporate credit re-repair.

H5: Leverage weakens the positive impact of tournament incentives on corporate credit re-repair.

## 4 Research design

### 4.1 Sample and data

To investigate the relationship between tournament incentives and corporate credit repair and re-repair, we collected data from 2009 to 2023 of A-shared firms listed on the Shenzhen and Shanghai stock exchanges. Consistent with previous studies, we excluded financial industry firms [62], firms under special treatment [63], and firms with one or more instances of missing data, and we winsorized all the relevant variables of interest at a 1% level. The primary data used to assess corporate credit repair and re-repair was manually gathered from the official Qichacha website.

### 4.2 Measures

#### 4.2.1 Independent variable.

***Corporate credit repair and re-repair.*** Corporate dishonest behavior encompasses bond defaults, labor arbitration, dishonesty listings, failed inspections, government inquiries, tax arrears, environmental penalties, bankruptcy reorganizations, operational irregularities, stock freezes, administrative penalties, enforcement actions, consumption restrictions, and blacklisting. Corporate credit repair (*CRP*) is the process by which firms, subject to administrative penalties or inclusion on a joint disciplinary blacklist, actively correct their discreditable actions within a specified timeframe, in accordance with prescribed conditions and procedures. Successfully rectifying these behaviors allows the firm to mitigate the adverse effects of prior misconduct on its credit supervision and evaluation [15]. Specifically, corporate credit repair (*CRP*) is defined as the process by which a firm, having engaged in dishonest behavior, complies with mandated regulatory procedures to restore its credit standing. In such instances, the variable *CRP* is assigned a value of 1; otherwise, the value is 0. Corporate credit re-repair (*CRRP*) refers to situations in which a firm engages in recurrent misconduct but consistently undertakes timely corrective actions to rectify each infraction. In these cases, *CRRP* is likewise assigned a value of 1; otherwise, the value is 0. To mitigate potential identification bias stemming from the inclusion of firms that have never exhibited dishonest conduct, the sample is restricted to those firms that have committed at least one misconduct during the observation period. The dependent variable thus reflects whether the firm undertook credit repair or re-repair following instances of misconduct. This sampling strategy ensures that the empirical analysis focuses on the reactions of enterprises after the occurrence of dishonesty, rather than the determinants of dishonesty in general. Furthermore, given the operationalization of the dependent variables, the central research question addressed in this study is whether tournament incentives (*TI*) influence firms' propensity to engage in credit repair or re-repair after misconduct has occurred, rather than whether such incentives affect the initial incidence of dishonest behavior. This identification strategy reduces endogeneity stemming from inherent behavioral tendencies, thereby enabling a sharper focus on how tournament incentives affect firms' choices regarding credit repair and re-repair.

#### 4.2.2 Dependent variable.

***Tournament incentive*** (*TI*). Based on previous research [7,8,31], we use the difference between total CEO compensation and the median value of total VP compensation, calculated below, to measure the strength of the tournament incentives. More specifically, *Tournament incentives* = Ln (total CEO compensation- median value of total VP compensation). The VPs in our sample are all non-CEO VPs, specifically vice presidents, deputy general managers, chief operating officers, chief financial officers, assistant managers, and other managers.

#### 4.2.3 Moderator variable.

***Firm age*** (*Age*) is measured by the natural logarithm of the firm's age. ***Firm size*** (*Size*) is measured by the natural logarithm of total assets [8]. ***CEO shareholding*** (*CEO shareholding*) is measured by the proportion of a company's outstanding shares owned by the CEO [64,65]. ***Leverage*** (*Lev*) is measured as total liabilities divided by total assets [66].

#### 4.2.4 Control variables.

Other control variables selected in this paper are defined as follows. We include Ownership (an indicator variable that equals 1 if the firm is state-owned and 0 otherwise), ROA (Net profit divided by average balance of total assets), DUAL (If the chairman and general manager are the same person, the value is 1, otherwise it is 0), Inventory ratio (Inventory balance at the end of the year divided by total assets), Fixed assets ratio (Fixed assets divided by total assets), Percentage of independent directors (Ratio of independent directors to total number of directors), Big Four International Auditing (The company is audited by one of the Big Four international accounting firms, and the value is 1; otherwise, the value is 0). In addition, this article also controls for firm and year fixed effects. The specific variable definitions are presented in Table 1.

**Table 1:** Variable definitions.

Name	Symbol	Definition
Corporate credit repair	*CRP*	If a firm undertakes credit repair, the variable takes the value of 1; otherwise, it is assigned a value of 0.
Corporate credit re-repair	*CRRP*	If a firm undertakes credit re-air, the variable takes the value of 1; otherwise, it is assigned a value of 0.
Tournament incentive	*TI*	Ln (total CEO compensation- median value of total VP compensation)
Firm age	*Age*	the natural logarithm of the firm's age
Firm size	*Size*	the natural logarithm of total assets
CEO shareholding	*CEO shareholding*	the proportion of a company's outstanding shares owned by the CEO
Leverage	*Lev*	total liabilities divided by total assets
Ownership	*SOE*	the firm is state-owned and 0 otherwise
ROA	*ROA*	Net profit divided by average balance of total assets
DUAL	*Dual*	If the chairman and general manager are the same person, the value is 1, otherwise it is 0
Inventory ratio	*Inv*	Inventory balance at the end of the year divided by total assets
Fixed assets ratio	*Fixed*	Fixed assets divided by total assets
Percentage of independent directors	*Indep*	Ratio of independent directors to total number of directors
Big Four International Auditing	*Big4*	The company is audited by one of the Big Four international accounting firms, and the value is 1; otherwise, the value is 0

The variable description and descriptive statistics of this article are shown in Table 2 below.

**Table 2:** Descriptive statistics of main variables.

Panel A:					
Variable	N	Mean	SD	Min	Max
Corporate credit repair (*CRP*)	19390	0.162	0.368	0	1
Tournament incentive (*TI*)	19390	12.74	1.384	3.778	17.36
CEO shareholding (*CEO shareholding*)	19390	0.044	0.106	0	0.790
Firm age (*Age*)	19390	2.958	0.340	1.099	3.689
Firm size (*Size*)	19390	22.47	1.291	19.31	26.45
Leverage (*Lev*)	19390	0.442	0.200	0.028	0.934
Ownership (*SOE*)	19390	0.381	0.486	0	1
ROA (*ROA*)	19390	0.041	0.062	−0.578	0.220
DUAL (*Dual*)	19390	0.266	0.442	0	1
Inventory ratio (*Inv*)	19390	0.146	0.137	0	0.772
Fixed assets ratio (*Fixed*)	19390	0.210	0.157	0.002	0.765
Percentage of independent directors (*Indep*)	19390	0.374	0.052	0.250	0.600
Big Four International Auditing (*Big4*)	19390	0.075	0.264	0	1
**Panel B:**					
**Variable**	**N**	**Mean**	**SD**	**Min**	**Max**
Corporate credit re-repair (*CRRP*)	11072	0.200	0.400	0	1
Tournament incentive (*TI*)	11072	12.75	1.393	3.778	17.17
CEO shareholding (*CEO shareholding*)	11072	0.037	0.099	0	0.675
Firm age (*Age*)	11072	2.970	0.339	1.099	3.689
Firm size (*Size*)	11072	22.56	1.301	19.31	26.45
Leverage (*Lev*)	11072	0.455	0.199	0.028	0.934
Ownership (*SOE*)	11072	0.421	0.494	0	1
ROA (*ROA*)	11072	0.040	0.059	−0.578	0.220
DUAL (*Dual*)	11072	0.249	0.433	0	1
Inventory ratio (*Inv*)	11072	0.151	0.142	0	0.772
Fixed assets ratio (*Fixed*)	11072	0.215	0.160	0.002	0.765
Percentage of independent directors (*Indep*)	11072	0.373	0.053	0.250	0.600
Big Four International Auditing (*Big4*)	11072	0.088	0.283	0	1

### 4.3 SHAP values

In this study, we employed SHAP (Shapley Additive Explanation) values both to evaluate feature importance and reveal potential nonlinearities and interaction effects among variables. This capability is particularly valuable for interpreting complex dependencies, such as the nonlinear threshold effects discussed later in this study, and providing a complementary perspective to traditional regression analyses. SHAP values are widely used for assessing feature importance in predictive models, as they measure the marginal contribution of a feature to the model's prediction outcome after incorporating that feature into the model [67]. SHAP values offer a detailed understanding of the decision-making processes within the model, enabling the identification of critical features, and they can also serve as a diagnostic tool for troubleshooting model performance issues. The SHAP values generated provide a measure of each feature's impact on the model's predictions for specific data points. Higher positive SHAP values indicate that a feature positively influences the prediction, while higher negative values reflect that a feature negatively influences the prediction. This allows for a comprehensive and impartial interpretation of the significance of each feature. SHAP values are model-agnostic, meaning they can be applied across various types of classifiers, providing a consistent evaluation approach. In this study, we implemented SHAP values using the SHAP library in Python, as outlined by Lundberg and Lee (2017).

Additionally, random forests, comprising an ensemble of decision trees, are commonly used for classification tasks [68]. Random forests generate multiple decision trees by introducing randomness into the tree-building process. When new data is introduced, each tree makes an independent classification decision, and the final predicted label is determined by the majority vote across all trees. In this paper, we trained a predictive model using random forests to assess corporate credit repair and re-repair. We then utilized SHAP values to evaluate the importance of various features and explore their interactions within the model.

### 4.4 Regression model

Besides, to test our hypothesis that there is a positive relationship between tournament incentives and corporate credit repair and re-repair. The regression model is constructed as follows:


$$CRP_{i,t}=\beta_{\text{0}}+\beta_{\text{1}}TI_{i,t}+\beta_{\text{2}}Controls_{i,t}+Firm\_FE+Year\_FE+\varepsilon_{i,t}$$ (1)

$$CRRP_{i,t}=\beta_{\text{0}}+\beta_{\text{1}}TI_{i,t}+\beta_{\text{2}}Controls_{i,t}+Firm\_FE+Year\_FE+\varepsilon_{i,t}$$ (2)

Among them, *i* represents companies, *t* represents year, *CRP* is the variable of corporate credit repair, *TI* is the tournament incentive variable, *CRRP* is the variable of corporate credit re-repair, *Controls* is the control variables, *Firm fixed effects* (*Firm_FE*) is the firm fixed effect, *Year fixed effects* (*Year_FE*) is the year fixed effect, $\epsilon_{i,t}$ represents random errors.

## 5 Results

### 5.1 SHAP values

#### 5.1.1 Corporate credit repair.

We employ SHAP feature importance and SHAP summary plot. The SHAP feature importance is a visualization tool that enables us to understand the importance of each feature. This graph displays the features ranked according to the mean absolute SHAP value, reflecting their influence on model predictions. The SHAP summary plot is another powerful visualization tool that facilitates the interpretation of the contribution of each feature to the output of a black-box model.

**Fig 1:**
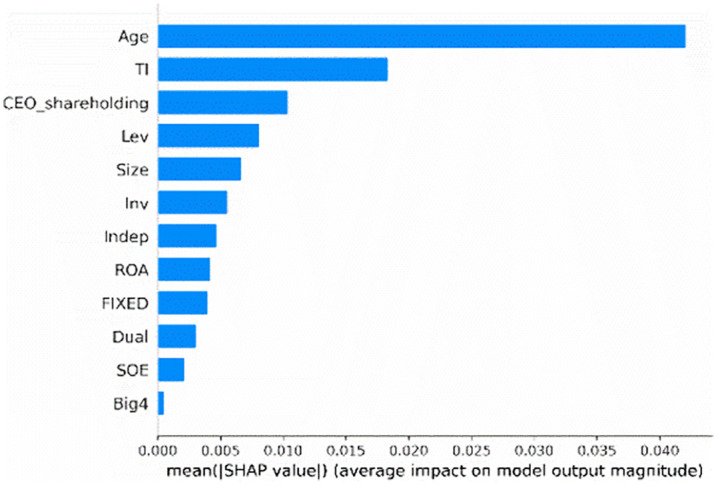
SHAP feature importance.

Fig 1 depicts the most prominent features that drive model predictions in our test sample. These features are ranked vertically based on their mean contribution on the prediction, with the most important feature positioned at the top. Specifically, firm age (*Age*), tournament incentives (*TI*), CEO shareholding (*CEO_shareholding*), and firm size (*Size*) are the most important features for predicting corporate credit repair, other features are also outlined in Fig 1, providing a comprehensive view of their contributions to the model's prediction. Then, focusing on the top-ranking feature, the prominence of firm age (*Age*) may capture systematic differences between mature and younger firms, such as in available resources, managerial experience, or inherent motivation for credit repair. Using the SHAP feature importance graph, we can focus on the most critical features, highlighting the variables with the greatest influence on model predictions.

**Fig 2:**
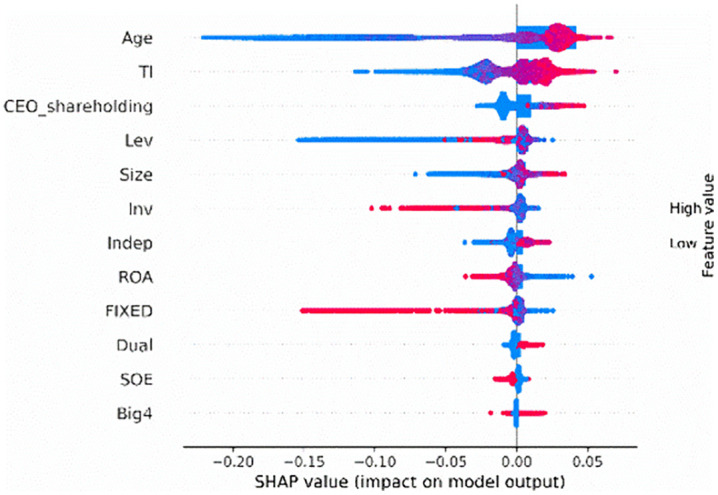
SHAP Summary Plot.

The SHAP summary plot (Fig 2) visualizes the shapley values of each feature on the x-axis, with the corresponding feature values on the y-axis. Each dot represents the feature value for a firm in the test sample. These dots are color-coded, ranging from red (high values) to blue (low values), representing the contribution of each feature to the model's output. A positive value suggests that the feature increases the likelihood of corporate credit repair, while a negative value reduces the likelihood. For instance, a high firm age (*Age*) emerges as the most influential feature in predicting corporate credit repair. Additionally, tournament incentives (*TI*) and CEO shareholding (*CEO_shareholding*) are also predictors of high credit repair. Then, focusing on the top-ranking feature, CEO shareholding (*CEO_shareholding*) emerges as an important predictive feature, likely capturing the alignment of managerial and shareholder interests, which in turn influences the commitment and proactive efforts of executives to restore corporate credit. By examining the SHAP summary plot, we can identify the most important features for predicting credit repair and their direction of impact.

Our analysis of the SHAP feature importance and SHAP summary plot reveal that tournament incentives are crucial for predicting corporate credit repair. Furthermore, it is essential to examine how firm age and CEO shareholding influence the likelihood of corporate credit repair.

**Fig 3:**
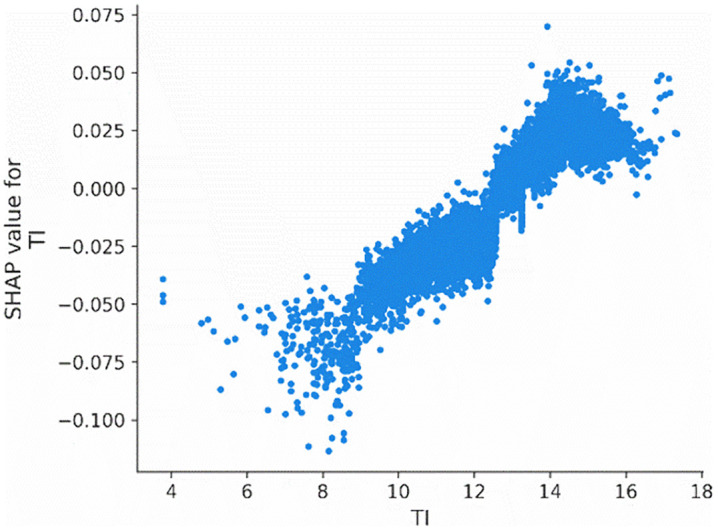
Main effect of TI.

We also employ the SHAP dependence plot to visualize the relationship between the most important features, firm age (*Age*), tournament incentives (*TI*), and CEO shareholding (*CEO_shareholding*), and their Shapley values. Fig 3 illustrates the influence of tournament incentives (*TI*) on the probability of corporate credit repair. Specifically, when the tournament incentives exceed 12, there is a significant increase in the probability of corporate credit repair.

**Fig 4:**
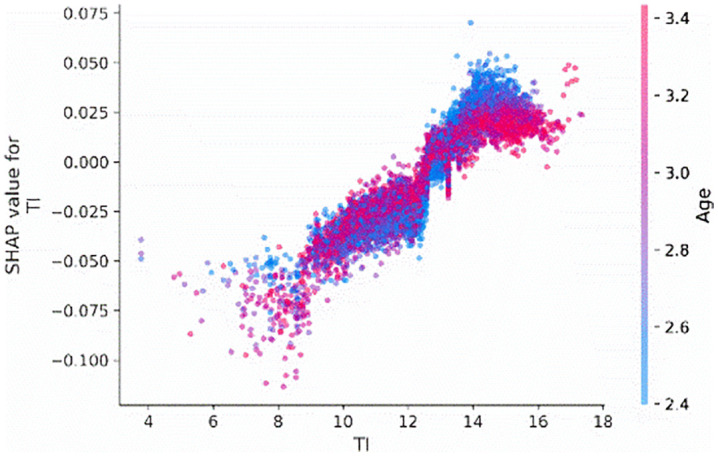
Interaction effect between TI and Age.

**Fig 5:**
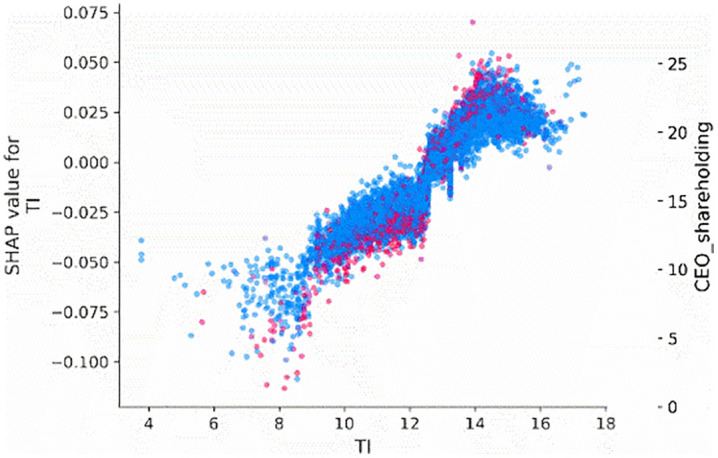
Interaction effect between TI and CEO_shareholding.

To examine the interaction effects between tournament incentives (*TI*) interact with firm age (*Age*) and CEO shareholding (*CEO_shareholding*), we visualize the interaction effects of these features on the tournament incentives. Figs 4 and 5 indicate that when the tournament incentives exceeding 12, the extent of its impact on predictions differed, as shown by the distribution of dots at the tournament incentive exceed 12. This means that other features affect the contribution of tournament incentives. According to Fig 4, the distribution representation of age values on the sample points, it can be concluded that the greater the tournament incentive, the portions colored red (denoting older firms) are situated below those colored blue, indicating that firm age diminishes the contribution of tournament incentives. This means that older firms tend to attenuate the positive impact of tournament incentives on corporate credit repair. Besides, according to Fig 5, based on the distribution of the red sample and the value color of CEO shareholding on the sample dots, it can be concluded that the higher the CEO shareholding ratio, the more positively tournament incentives will contribute. This means that the higher the CEO shareholding ratio, tends to increase the effect of tournament incentives on corporate credit repair.

#### 5.1.2 Corporate credit re-repair.

**Fig 6:**
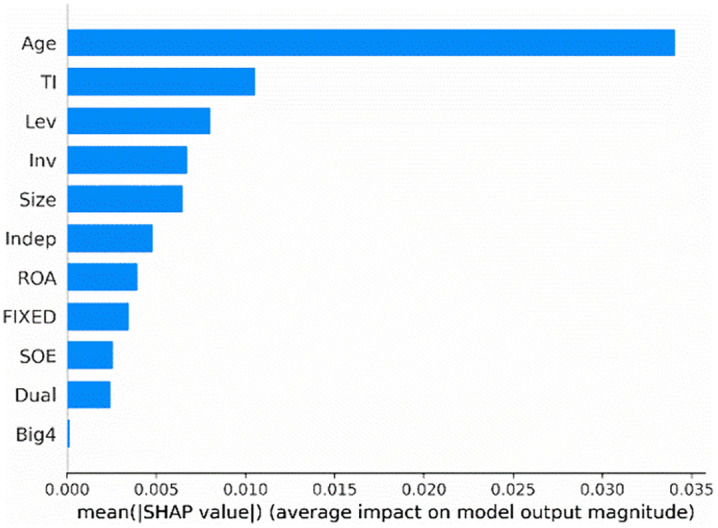
SHAP Feature Importance.

**Fig 7:**
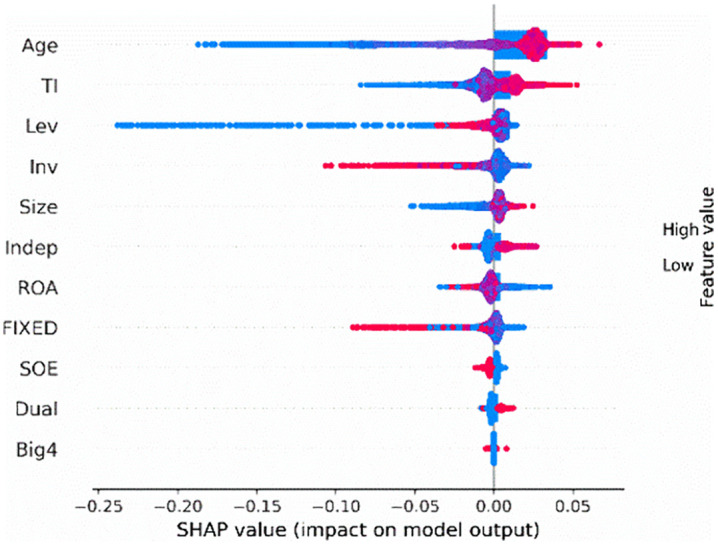
SHAP Summary Plot.

Fig 6 also highlights the key features driving model predictions in our test sample. Specifically, firm age (*Age*), leverage (*Lev*), tournament incentives (*TI*), and firm size (*Size*) are the most important features for predicting corporate credit re-repair, other features are also outlined in Fig 6, with a detailed overview of their importance. Focusing on the top-ranking feature, Firm size (*Size*) emerges as an important predictive feature, possibly capturing the benefits of larger firms' resources, governance capabilities, and incentive structures, which in turn affect their ability and effectiveness in executing corporate credit re-repair. The SHAP summary plot (Fig 7) illustrates the Shapley values of each feature on the x-axis and the corresponding feature values on the y-axis. For instance, low leverage (*Lev*) emerges as a key predictive feature in predicting corporate credit re-repair. Leverage (Lev) emerges as an important predictive feature, possibly indicating a firm's financial risk exposure and debt obligations, which in turn may influence its constraints and decision-making in executing corporate credit re-repair. Additionally, tournament incentives (*TI*) are also a predictor of high corporate credit re-repair.

Our analysis of the SHAP feature importance and SHAP summary plot reveals that tournament incentives are crucial for predicting corporate credit re-repair. It's also crucial to examine how firm size and leverage affect the chance of going corporate credit re-repair.

**Fig 8:**
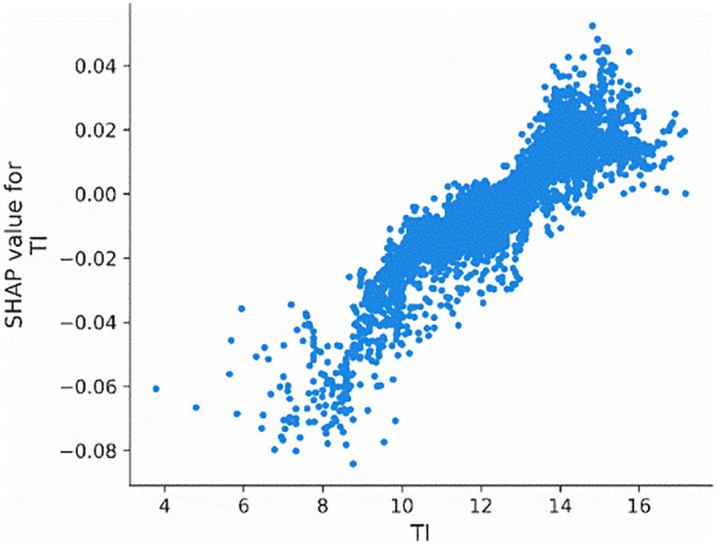
Main effect of TI.

Fig 8 depicts the effect of tournament incentives (*TI*) on the probability of corporate credit re-repair. Specifically, when the tournament incentives exceed 12, there is a significant increase in the probability of corporate credit re-repair.

**Fig 9:**
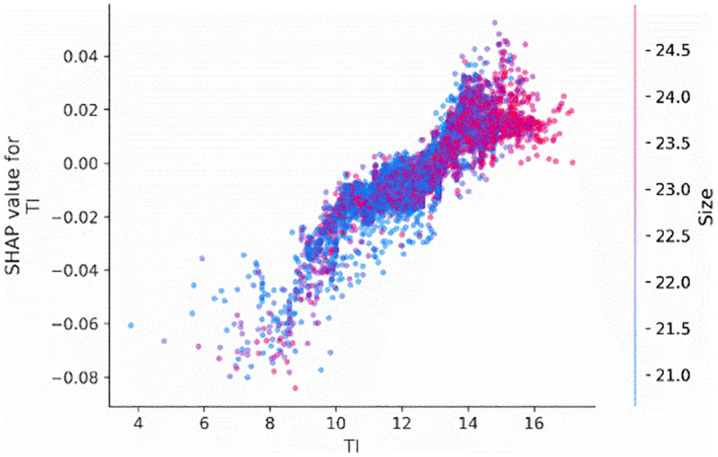
Interaction effect between TI and size.

**Fig 10:**
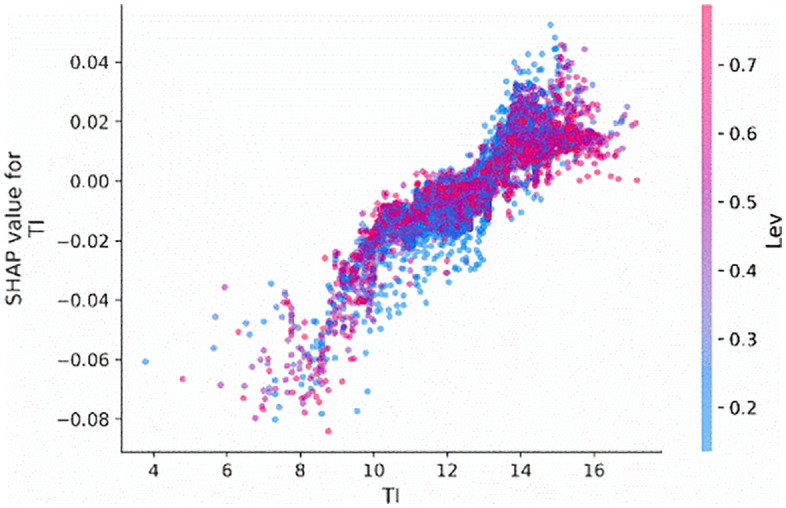
Interaction effect between TI and Lev.

To observe how tournament incentives (*TI*) interact with firm size (*Size*) and leverage (*Lev*) features, we visualize the interaction effects between tournament incentives and these firm characteristics. According to the distribution of Size values on the sample dots in Fig 9, the red section (representing larger firms) is positioned above the blue section. However, as the value of tournament incentives increases, the red section gradually shifts beneath the blue section. This indicates that the interaction effect between tournament incentives and firm size weakens with an increase in tournament incentives, causing the interaction coefficient to turn negative. This suggests that the larger the firm, the more likely it is to diminish the positive effect of tournament incentives on corporate credit re-repair. Similarly, based on Fig 10, it can also be observed that as the firm's leverage increases, it is more likely to weaken the positive impact of tournament incentives on corporate credit re-repair.

### 5.2 Regression results

#### 5.2.1 Baseline regression results.

In addition to the methods mentioned above, we also employed econometric analysis to further support our findings. Columns (1) and (2) of Table 3 present the result of the model regression on the relationship between tournament incentives and corporate credit repair and re-repair. As shown in Columns (1), the coefficient of *TI* is significantly positive (beta = 0.008, p < 0.01), suggesting that as the intensity of tournament incentives between CEOs and non-CEO executives continues to escalate, it has the power to motivate corporate credit repair. This result is consistent with H1a. As shown in Columns (2), the coefficient of *TI* is significantly positive (beta = 0.008, p < 0.05), suggesting that as the intensity of tournament incentives between CEOs and non-CEO executives continues to escalate, it has the power to motivate corporate credit re-repair. This result is consistent with H1b.

**Table 3:** Result of regression.

	(1)	(2)
Variables	CRP	CRRP
TI	0.008^***^	0.008^**^
	(2.73)	(2.04)
Age	0.148^***^	0.214^***^
	(3.96)	(4.09)
Size	−0.013^*^	−0.021^**^
	(−1.92)	(−2.21)
ROA	0.053	0.045
	(0.87)	(0.50)
Lev	0.041	0.048
	(1.46)	(1.21)
SOE	0.015	−0.000
	(0.91)	(−0.01)
Dual	0.004	0.002
	(0.45)	(0.13)
Inv	−0.043	−0.091
	(−0.95)	(−1.50)
Fixed	0.019	0.020
	(0.50)	(0.39)
Indep	0.157^**^	0.173
	(2.00)	(1.59)
Big4	−0.022	−0.003
	(−0.89)	(−0.11)
Year_FE	Yes	Yes
Firm_FE	Yes	Yes
Constants	−0.175	−0.128
	(−1.09)	(−0.57)
Observations	19390	11072
R^2^	0.023	0.026

Note: *, **, and *** denote that a statistic is significant at the 10%, 5%, and 1% levels, respectively.

### 5.2.2 Robustness tests

In this section, we take several steps to ensure robustness in reported results of tournament incentives and corporate credit repair and re-repair.

First, we re-estimate the regression using the difference between total CEO compensation and the mean value of total VP compensation as an alternative proxy for tournament incentives in the robustness analysis [69]. The relationship between tournament incentives and corporate credit repair and re-repair remains positive, and the result is shown in columns (1) and (2) of Table 4. We observe that the regression coefficient of Tournament incentives is significantly positive at the 5% level. This indicates that tournament incentives have a positive impact on corporate credit repair and re-repair. This can prove that our benchmark regression results are robust.

**Table 4:** Replace explanatory variables.

	(1)	(2)
Variables	CRP	CRRP
TI	0.012^***^	0.009^*^
	(3.36)	(1.71)
	(−1.33)	(−0.57)
Control variables	Yes	Yes
Year_FE	Yes	Yes
Firm_FE	Yes	Yes
Observations	17972	10294
R^2^	0.023	0.026

Note: *, **, and *** denote that a statistic is significant at the 10%, 5%, and 1% levels, respectively.

Second, given that the dependent variable is binary, we assess the robustness of our findings through logistic regression analysis. The results of the logistic regression analysis are reported in columns (1) and (2) of Table 5. The regression result of the impact of tournament incentives on corporate credit repair and re-repair, which is positive and significant at the 1% and 5% levels, supporting H1a and H1b. This can also prove that our benchmark regression results are basically robust.

**Table 5:** Logit model.

	(1)	(2)
Variables	CRP	CRRP
TI	0.071^***^	0.059^*^
	(2.70)	(1.90)
Control variables	Yes	Yes
Year_FE	Yes	Yes
Firm_FE	Yes	Yes
Observations	17034	10351
R^2^	0.095	0.076

Note: *, **, and *** denote that a statistic is significant at the 10%, 5%, and 1% levels, respectively.

Third, considering the differences in compensation structures across industries, we further control for industry fixed effects to ensure the robustness of our results. As shown in Table 6, the estimated coefficient of tournament incentives remains significantly positive.

**Table 6:** control for industry fixed effects.

	(1)	(2)
Variables	CRP	CRRP
TI	0.008^***^	0.009^**^
	(2.70)	(2.07)
Control variables	Yes	Yes
Year_FE	Yes	Yes
Firm_FE	Yes	Yes
Ind_FE	Yes	Yes
Observations	19390	11072
R^2^	0.022	0.025

Note: *, **, and *** denote that a statistic is significant at the 10%, 5%, and 1% levels, respectively.

Fourth, considering that CEO age may influence the effectiveness of incentives—for instance, under the same level of tournament incentives, executives with promising promotion trajectories may respond differently than those with constrained advancement prospects—we further control for CEO age. The regression results (see Table 7) show that the estimated coefficient of tournament incentives remains significantly positive.

**Table 7:** Control for CEO age.

	(1)	(2)
Variables	CRP	CRRP
TI	0.008^***^	0.008^*^
	(2.69)	(1.81)
CEO age	−0.001	−0.001
	(−1.18)	(−1.37)
Control variables	Yes	Yes
Year_FE	Yes	Yes
Firm_FE	Yes	Yes
Observations	15411	9137
R^2^	0.026	0.029

Note: *, **, and *** denote that a statistic is significant at the 10%, 5%, and 1% levels, respectively.

Fifth, considering the potential bidirectional causality among the explanatory variables, control variables, and the dependent variable, this study adopts the standard approach of one-period lagging the dependent variable to further address potential endogeneity. This approach mitigates potential simultaneity concerns between tournament incentives and corporate credit repair and re-repair. As shown in Table 8, the coefficients of tournament incentives remain significantly positive, indicating that the conclusions drawn from the baseline regressions are consistent and robust.

**Table 8:** Lagged dependent variable.

	(1)	(2)
Variables	CRP	CRRP
TI	0.009^***^	0.011^**^
	(2.58)	(2.10)
Control variables	Yes	Yes
Year_FE	Yes	Yes
Firm_FE	Yes	Yes
Observations	14846	8548
R^2^	0.025	0.026

Note: *, **, and *** denote that a statistic is significant at the 10%, 5%, and 1% levels, respectively.

Sixth, the executive pay gap in Chinese listed firms is not random and is affected by corporate financial condition, corporate governance, and other firm characteristics. Those firm characteristics may also be associated with tournament incentives. The PSM approach is an appropriate method to address the endogeneity problem in executive compensation studies [70]. Therefore, to mitigate potential sample selection bias, we employ the propensity score for the presence of a substantial executive pay gap between executives. We introduce a new dummy, *Dummy_TI*. It equals 1 if the company pay gap is above the upper 50% percentile, otherwise 0. Then *Dummy_TI* is regressed on a set of variables capturing firm characteristics, including *Age, Size, SOE, ROA, Lev, SOE, Dual, Inv, Fixed*, *Indep* and *Big4*. We perform this logistics regression and the results are reported in Table 9, Panel A. Using the results estimated, we then calculate the propensity score and use the kernel matching method to construct a matched sample between the treatment group and control group. Then, the results of the regression analysis on the matched sample are reported in Panel B of Table 9. The coefficients on tournament incentive variables are still significantly positive. Therefore, our results remain robust after accounting for potential endogeneity.

**Table 9:** Propensity score matching.

Panel A: Summary statistics of matching criteria					
	Mean		Difference in means		
Variable	Treated	Control	t-stats		p-value
Age	3.048	3.045	0.420		0.674
Size	23.079	23.100	−0.770		0.441
ROA	0.052	0.049	2.140		0.032
Lev	0.466	0.469	−0.800		0.426
SOE	0.323	0.339	−1.590		0.113
Dual	0.282	0.281	0.070		0.942
Inv	0.163	0.161	0.620		0.533
Fixed	0.185	0.187	−0.670		0.506
Indep	0.374	0.375	−0.330		0.739
Big4	0.134	0.139	−0.720		0.470
**Panel B: Regression from PSM sample**					
**Regression**	**(1)**			**(2)**	
**Variables**	**CRP**			**CRRP**	
Dummy_TI	0.020**			0.024*	
	(2.16)			(1.83)	
Observations	19361			11050	
R^2^	0.023			0.026	
Control variables	Yes			Yes	
Year_FE	Yes			Yes	
Firm_FE	Yes			Yes	

Note: *, **, and *** denote that a statistic is significant at the 10%, 5%, and 1% levels, respectively.

## 6 Conclusion and discussion

Based on the data of Chinese A-share listed companies, we investigate the effects of tournament incentives on corporate credit remediation and re-repair, using the SHAP values and benchmark traditional econometric methods. We find that tournament incentives have a positive impact on corporate credit repair and re-repair. We further find that firm age weakens the positive impact of tournament incentives on corporate credit repair. CEO shareholding strengthens the positive impact of tournament incentives on corporate credit repair. Additionally, firm size weakens the positive impact of tournament incentives on corporate credit re-repair. Leverage weakens the positive impact of tournament incentives on corporate credit re-repair.

### 6.1 Theoretical contributions

First, we enrich the tournament literature by applying both SHAP values and benchmark traditional econometric methods to demonstrate a positive relationship between tournament incentives and corporate credit repair and re-repair. Although prior research has investigated tournament incentives in relation to corporate performance [18], risk-taking [31], fraud [7], cash holdings [26], innovation [23], and debt contracting [21]. Regarding corporate credit, previous studies have provided foundational insights for our understanding of corporate dishonest behavior from the perspective of tournament incentives [7,8]. However, little attention has been paid to how such incentives may affect a firm's efforts to repair or re-repair its creditworthiness following dishonest behavior. Our findings reveal that tournament incentives promote the credit repair and re-repair of Chinese enterprises, thereby contributing to tournament literature by enhancing our understanding of the links between corporate compensation policies and corporate credit repair and re-repair.

Second, our study contributes to the corporate credit literature by explicitly addressing a critical gap: prior research has primarily focused on the antecedents of corporate dishonesty while largely neglecting post-dishonesty remediation mechanisms. Previous studies have explored the antecedents of corporate dishonest behavior from various perspectives, encompassing executive team characteristics [9,10], board characteristics [11], independent director characteristics [12], executive compensation [6], executive backgrounds [13], and equity structure [14]. However, these studies primarily focus on whether these factors can mitigate (increase) the occurrence of corporate dishonest behavior, ignoring how to remediate such behaviors post-occurrence. Moreover, in the context of China, where financial market regulation and enforcement are relatively weaker compared to common law countries, such behavioral issues could be particularly severe [6]. Our study provides both theoretical and empirical evidence that tournament incentives positively influence corporate credit repair and re-repair. In doing so, it extends the corporate credit literature beyond an antecedent-focused perspective and fills the previously overlooked research gap concerning post-occurrence remediation.

Third, our research contributes to the corporate governance literature by examining how the interaction between tournament incentives and CEO shareholding, as well as firm age, relates to corporate credit repair, and how the interaction between tournament incentives, corporate size, and leverage corresponds with corporate credit re-repair. The literature on corporate governance highlights that incentive mechanisms are instrumental in mitigating agency problems [28]. Yet, the moderating roles of executive and firm characteristics in the context of credit repair and re-repair. Our research represents a significant preliminary effort to fill these research gaps by exploring the moderating roles of CEO characteristics and firm attributes in the relationship between tournament incentives and corporate credit repair and re-repair, and it shows that heterogeneity in these characteristics may yield non-synergistic effects. This can also further explore the boundary conditions between tournament incentives and corporate credit repair and re-repair.

### 6.2 Practical implications

First, our findings are vital for boards of directors seeking to optimize more effective executive compensation decisions. Compensation structures are a fundamental tool for addressing agency problems and aligning managerial incentives with shareholder interests. By encouraging competition among executives, tournament incentives can enhance CEO effort and managerial efficiency, ultimately promoting better corporate performance. Since performance-based promotions are often tied to compensation, CEOs motivated by tournament incentives are more likely to implement strategic initiatives to enhance corporate reputation, including corporate credit repair and re-repair. Boards should therefore recognize the positive role of tournament incentives and adjust incentive schemes judiciously, particularly for firms seeking to restore credibility after reputational damage.

Second, our findings provide investors with a framework to assess investment risks based on the company's compensation structures. Given the increasing global emphasis on compensation disclosure—as evidenced by regulatory frameworks in the U.S., U.K., and other countries that require public firms to report CEO pay and pay ratios—our study suggests that such transparency can serve as an important signal. Investors can utilize insights into compensation structures, particularly the presence and intensity of tournament incentives, to evaluate a firm's governance practices and anticipate its potential for reputation restoration. This enables more prudent and discerning investment choices.

Third, our findings hold crucial significance for governments seeking to supervise, present, and govern corporate credit repair and re-repair. Corporate dishonesty undermines investor confidence and poses systemic risks to the capital market. Thus, it is imperative to strengthen the social credit system and provide pathways for firms to restore creditworthiness and reintegrate into the market. Supporting mechanisms for credit repair and re-repair can help stabilize business operations, encourage lawful behavior, and stimulate market dynamism. Furthermore, our findings underscore the importance of developing a government-led credit supervision framework, which can mitigate dishonest practices, support corporate turnaround efforts, and cultivate a more resilient and credible business ecosystem.

Fourth, our study provides valuable guidance for corporate governance, particularly for internal compensation committees. Beyond motivating short-term performance, executive pay structures exert a decisive influence on a firm's long-term reputation and risk culture. Boards and compensation committees should look past narrow financial metrics and incorporate non-financial levers that encourage ethical conduct and responsible credit management. By aligning tournament incentives with credit restoration goals, firms can foster a governance environment that values integrity and sustainable reputation—not just immediate outcomes. This internal focus helps companies build resilience, reduce recurrent misconduct, and preserve long-term stakeholder trust.

### 6.3 Limitations and future research

First, the institutional and cultural context of China, characterized by high power distance, provides a distinctive context in which tournament incentives can wield a more pronounced effect on executive behavior. Additionally, institutional weaknesses, such as underdeveloped legal systems and regulatory gaps, foster a greater incidence of corporate malfeasance, such as corporate financial fraud across the nation [66], thereby creating fertile ground for examining the impact of tournament incentives on credit repair and re-repair. However, this context-specific setting constrains the generalizability of our findings. Future research should reproduce and substantiate these findings in countries or regions with lower power distance and stronger institutional frameworks, to assess the degree to which cultural and institutional variables moderate the effects of tournament incentives.

Second, this research insufficiently considers CEO characteristics, which may profoundly influence how tournament incentives influence managerial behavior. While agency theory views CEOs as rational and self-interested agents [28], stewardship theory posits that intrinsic motivation, organizational identification, and personal values [71] may drive CEOs to prioritize the interests of shareholders [72]. As such, future studies might explore potential moderating variables such as CEO awards [10] and Military Background [13] to more precisely delineate the boundary conditions of the relationship between tournament incentives and corporate credit repair or re-repair.

Third, the intrinsic obscurity of corporate dishonest behaviors presents a measurement challenge. Due to the concealed nature of misconduct, certain firms deemed credit-restored may have covertly resumed malfeasant conduct undetected. This misclassification could affect the robustness of our findings. Future research should employ more sophisticated detection methodologies to improve measurement accuracy. In addition to machine learning algorithms for fraud prediction, forensic accounting data, or whistleblower disclosures, researchers might leverage natural language processing techniques to extract soft information from news media, analyst reports, or regulatory filings. Such approaches can provide additional insights into corporate reputation and the effectiveness of credit repair measures, complementing existing measurement methods and thereby enhancing the robustness and credibility of empirical findings.
